# Protein phosphatase 2A as a therapeutic target in inflammation and neurodegeneration

**DOI:** 10.1016/j.pharmthera.2019.05.016

**Published:** 2019-09

**Authors:** Andrew R. Clark, Michael Ohlmeyer

**Affiliations:** a Institute of Inflammation and Ageing, College of Medical and Dental Sciences, University of Birmingham, Edgbaston, Birmingham, B15 2TT, United Kingdom; b Atux Iskay LLC, Plainsboro, NJ, USA

**Keywords:** Protein phosphatase 2A, Inflammation, Cancer, Neurodegeneration, Alzheimer’s disease, Multiple sclerosis, AD, Alzheimer’s disease, AP-1, activator protein 1, BBB, blood-brain barrier, CCR4/NOT, carbon catabolite repressor 4/never on TATA-less, CIP2A, cancerous inhibitor of PP2A, CNS, central nervous system, DC, dendritic cell, EAE, experimental autoimmune encephalopathy, HEAT, Huntingtin - Elongation factor - A subunit of PP2A - Target of rapamycin domain, IκBα, α inhibitor of NF-κB, IKK, IκBα kinase, IL, interleukin, IRF, interferon-regulatory factor, JNK, cJun N-terminal kinase, LCMT-1, leucine carboxymethyl transferase 1, LUBAC, linear ubiquitin chain assembly complex, MAPK, mitogen-activated protein kinase, MK2, MAPK-activated kinase 2, MKK, MAPK kinase, MS, multiple sclerosis, MyD88, myeloid differentiation factor 88, NF-κB, nuclear factor κ enhancer of activated B cells, NFT, neurofibrillary tangles, PAD, PP2A-activating drug, PAMP, pathogen-associated molecular pattern, PME-1, protein phosphatase methylesterase 1, PP2A, protein phosphatase 2A, PRR, pattern recognition receptor, ROS, reactive oxygen species, S1P, sphingosine-1-phosphate, S1PR, S1P receptor, SphK, sphingosine kinase, STRN, striatin, TAB, TAK1 binding protein, TAK1, transforming growth factor β-activated kinase, TGFβ, transforming growth factor β, TIRAP, Toll/interleukin 1 receptor domain-containing adaptor protein, TLR, Toll-like receptor, TNF, tumor necrosis factor, TRAF, TNF receptor interacting factor, TRAM, Toll/interleukin 1 receptor domain-containing adaptor molecule, TRIF, Toll/interleukin 1 receptor domain-containing adaptor inducing interferon β, TTP, tristetraprolin, UVB, ultraviolet B

## Abstract

Protein phosphatase 2A (PP2A) is a highly complex heterotrimeric enzyme that catalyzes the selective removal of phosphate groups from protein serine and threonine residues. Emerging evidence suggests that it functions as a tumor suppressor by constraining phosphorylation-dependent signalling pathways that regulate cellular transformation and metastasis. Therefore, PP2A-activating drugs (PADs) are being actively sought and investigated as potential novel anti-cancer treatments. Here we explore the concept that PP2A also constrains inflammatory responses through its inhibitory effects on various signalling pathways, suggesting that PADs may be effective in the treatment of inflammation-mediated pathologies.

## 1 Introduction

Protein phosphorylation, the reversible, covalent addition of phosphate groups to serine, threonine or tyrosine residues, is a rapid and efficient mechanism for modulating protein function. This post-translational odification alters the charge, local shape and global conformation of substrate proteins, influencing their interactions with other molecules, and modulating their subcellular localization, stability or function. The human genome encodes more than 500 kinases that catalyze protein phosphorylation, and fewer than 200 protein phosphatases that catalyze the reverse reaction. However, these numbers may be misleading. In some cases substrate specificity can be conferred by regulatory subunits distinct from the proteins that possess catalytic activity. Duplication and evolutionary diversification of regulatory subunits can therefore greatly expand the functional roles of kinases or phosphatases without a parallel increase in the number of kinase- and phosphatase-encoding genes. Such diversification appears to have been particularly important in the evolution of protein phosphatase(s) 2A (PP2A), the subject of this review.

Since protein phosphorylation has profound effects on every aspect of cell biology, precise balance between the activities of kinases and phosphatases is required for the proper regulation of cell function. Strikingly, such balance is not seen in scientific literature, where published articles with “kinase” outnumber those with “phosphatase” in their titles by a factor of almost ten to one. There are several possible explanations for such bias. By definition phosphorylation is an energy-dependent process requiring the consumption of ATP. For reasons of energy economy, evolution may have favored use of protein phosphorylation as a means of response to perturbation, and dephosphorylation as a means of restoring or maintaining equilibrium. This generalization seems broadly true, although many readers will readily think of exceptions. In this perspective, phosphatase activity may be seen as rather non-specific, unregulated, and consequently not very interesting as a subject of study. Recent advances suggest that these are all misconceptions. Inhibition of kinases to prevent harmful cell activation is more conceptually and practically straightforward than stimulation of phosphatases to promote restoration of homeostasis. Much like the scientific literature, patent literature demonstrates strong bias towards targeting of kinases rather than phosphatases. In this review we argue that improved understanding of cell signalling may open up new opportunities to exert therapeutic effects via activation of the phosphatase(s) PP2A.

## 2 The regulation of PP2A function

### 2.1 PP2A subunits and regulators

**Fig. 1:**
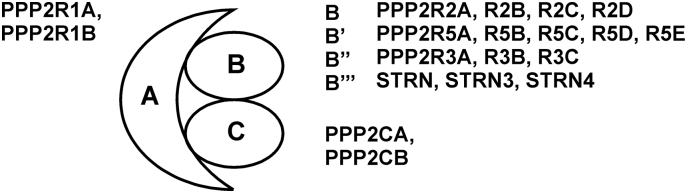
Composition of the PP2A holoenzyme. Systematic names are indicated for scaffolding (A), regulatory (B) and catalytic (C) protein subunits. Alternative names are indicated in Table 1.

Readers may have noticed our reference to “the phosphatase(s) PP2A”. The reason for this form of words is that PP2A is not a single entity but a family of heterotrimeric holoenzymes. The active enzyme is a complex containing a 65 kD scaffolding subunit (A), a 36 kD catalytic subunit (C) and a specificity-determining subunit of variable size (B) (Haesen, Sents, Lemaire, Hoorne, & Janssens, 2014; Lambrecht, Haesen, Sents, Ivanova, & Janssens, 2013). Two distinct and functionally non-redundant genes, PPP2R1A and PPP2R1B, encode α and β scaffolding subunits of PP2A. These proteins contain fifteen repeats of a 39 amino acid structural motif known as the HEAT domain, being present in **H**untingtin, **E**longation factor, the **A** subunit of PP2A, and **T**arget of Rapamycin. The HEAT domains stack one above another to form an extended hook structure, with somewhat flexible loops between them, and a highly flexible hinge between the twelfth and thirteenth repeats (Cho & Xu, 2007; Jiang et al., 2013; Y. Xu et al., 2006). There are also two distinct genes, PPP2CA and PPP2CB, encoding α and β catalytic subunits. In the majority of adult tissues the α isoforms of both scaffolding and catalytic subunits appear to be predominant. Functional complexity and diversity of PP2A therefore arise largely via the B subunits, which determine both the subcellular localization and substrate specificity of individual heterotrimers (Haesen et al., 2014; Lambrecht et al., 2013). B subunits are encoded by four families of genes, comprising a total of fifteen genes. These are the B (*PPP2R2*) family, with four members; the B’ (*PPP2R5*) family, with five members; the B” (*PPP2R3*) family, with three members; and the Striatin (*STRN*) family, with three members (Fig. 1). Several of these genes generate different products via alternative splicing. The four families of proteins are structurally distinct from one another, and their accommodation within the holoenzyme is permitted by the intrinsic flexibility of the A (scaffolding) subunit. In this review we will adhere to the systematic names for B subunits. Alternative names are listed in Table 1.

**Table 1:** Systematic and alternative names of PP2A protein subunits. Based on Human Genome Organization Gene Nomenclature Committee and Genecards (https://www.genecards.org). Asterisks indicate where different protein products are known to be generated via alternative splicing of primary transcripts.

Subunit type	Sub-type	Systematic name	Alternative names
A (scaffolding)		PPP2R1A	PR65α, PR65A, PP2A-Aα, MRD36
		PPP2R1B	PR65β, PR65B, PP2A-Aβ
B (regulatory)	B	PPP2R2A	Bα, PR55α, B55α
		PPP2R2B*	Bβ, PR55β, B55β, SCA12
		PPP2R2C	Bγ, PR55γ, B55γ
		PPP2R2D	Bδ, PR55δ, B55δ, KIAA1541
	B”	PPP2R3A*	B”α, PR130, PR72
		PPP2R3B*	B”β, PR70, PR48
		PPP2R3C	B”γ, C14orf10, G5PR
	B’	PPP2R5A	B’α, PR61α, B56A, B56α
		PPP2R5B	B’β, PR61β, B56B, B56β
		PPP2R5C*	B’γ, PR61γ, B56G, B56γ
		PPP2R5D*	B’δ, PR61δ, B56D, B56δ, MRD35
		PPP2R5E*	B’ε, PR61ε, B56E, B56ε
	B”’Striatin	STRN	B”’α, PPP2R6A, PR93, STRN1
		STRN3	B”’β, PPP2R6B, PR110, SG2NA
		STRN4	B”’γ, PPP2R6C, zinedin, ZIN
C (catalytic)		PPP2CA	PP2A-Cα, PP2CA, PP2Aα
		PPP2CB	PP2A-Cβ, PP2CB, PP2Aβ

PP2A profoundly influences all aspects of cell biology, therefore its function is tightly regulated at several levels. As reviewed extensively elsewhere (Janssens, Longin, & Goris, 2008; Sents, Ivanova, Lambrecht, Haesen, & Janssens, 2013), assembly of holoenzymes is strictly controlled to prevent the formation of catalytically active complexes lacking correct substrate specificity. Unpartnered catalytic subunits are subject to ubiquitination and proteasomal degradation. The protein α4 (otherwise known as Immunoglobulin Binding Protein 1 or IGBP1) both stabilizes and inactivates free C subunits, thereby regulating their availability for assembly into holoenzymes (Kong, Ditsworth, Lindsten, & Thompson, 2009). The ATP-dependent chaperone phosphotyrosyl phosphatase activator (encoded by *PPP2R4*) is required for correct folding of the catalytic subunit and incorporation of manganese ions at the catalytic site (Cho & Xu, 2007; Guo et al., 2014; Jordens et al., 2006). The catalytic subunit is also post-translationally modified by methylation of the free carboxyl group of the C-terminal leucine residue, Leu309. This unusual modification is performed by leucine carboxymethyl transferase 1 (LCMT-1), and can be reversed by protein phosphatase methylesterase 1 (PME-1). Assembly of certain holoenzyme complexes, particularly those containing B subunits of the PPP2R2 family, is critically dependent on methylation of the C subunit (Cho & Xu, 2007). It has been reported that phosphorylation of the adjacent tyrosine residue Tyr307 antagonizes Leu 309 methylation, rendering PP2A subject to control by protein tyrosine kinases and phosphatases (Chen, Martin, & Brautigan, 1992; Chen, Parsons, & Brautigan, 1994; Liu et al., 2008; Zonta et al., 2015). However, Tyr307 phosphorylation is not supported by unbiased phospho-proteomic studies (Hornbeck et al., 2015) and an antibody commonly used to detect this modification is unreliable (Ogris, Sontag, Wadzinski, & Narla, 2018). Mechanisms of cross-talk between PP2A and tyrosine kinases/phosphatases therefore require clarification.

Protein kinase A-mediated phosphorylation of the B subunit PPP2R5D increases the activity of PP2A against certain phosphoprotein substrates (Ahn et al., 2007; Dodge-Kafka et al., 2010; Ranieri, Kemp, Burgoyne, & Avkiran, 2018; Yu & Ahn, 2010). According to a database of unbiased, high throughput proteomic studies (Hornbeck et al., 2015), other members of the PPP2R5 family also appear to be phosphorylated within similar, highly charged N-terminal domains, suggesting that they too may be regulated via phosphorylation by cyclic AMP-dependent (or other) kinases. Perusal of the same database indicates many other well-documented but unstudied post-translational modifications of B subunits. For example, members of the STRN family appear to be extensively phosphorylated. There is also consistent evidence for the acetylation of both PPP2R2A and PPP2R2D at lysine residues near to their C-termini. At least in the plant *Arabidopsis thaliana*, PP2A associates with protein acetylases and deacetylases in the vicinity of microtubules, where it may participate in the control of cytoskeletal dynamics (Tran et al., 2012). Although some studies have hinted at crosstalk between phosphorylation and acetylation of PP2A substrates (Nunbhakdi-Craig et al., 2007), consequences of acetylation of PP2A subunits have not been investigated as far as we know.

The cellular repertoire of PP2A targets is governed by the expression of B subunits, which is under developmental and tissue-specific control (Haesen et al., 2014; Reynhout & Janssens, 2019). In the transformed T cell line Jurkat, engagement of the T cell receptor caused up-regulation of *PPP2R5C* mRNA and the corresponding protein (Breuer et al., 2014). *PPP2R2A* mRNA and its protein product were up-regulated by interferon treatment of primary human macrophages (Schott et al., 2018). Our own, unpublished observations revealed both increases and decreases in expression of various B subunits mRNAs after lipopolysaccharide (LPS) stimulation of primary human and mouse macrophages. We hypothesise that such changes result in stimulus-dependent modulation of PP2A activity and/or substrate specificity. However, the role of regulated B subunit expression in signal transduction has not been extensively studied.

Several endogenous proteins have been shown to negatively regulate PP2A function. Both PME-1 and α4 could be considered as PP2A inhibitors, although this does not do justice to their complex and essential roles in assembly of the holoenzyme. The best characterized inhibitors are ANP32A (Acidic Nuclear Phosphoprotein 32A, otherwise known as PP2A inhibitor 1); the closely related protein ANP32E; SET (also known as PP2A inhibitor 2); and CIP2A (cancerous inhibitor of PP2A). SET binds to PPP2CA and inhibits its phosphatase activity (Arnaud et al., 2011). In contrast, CIP2A inhibits holoenzyme activity by binding to PPP2R5A or PPP2R5C components (Wang et al., 2017), its specificity for other B subunits remaining unclear. Thus the interaction of inhibitor proteins with PP2A provides a level at which phosphatase activity can be further controlled by cellular signaling pathways.

### 2.2 Sphingolipid metabolism and the control of PP2A function

**Fig. 2:**
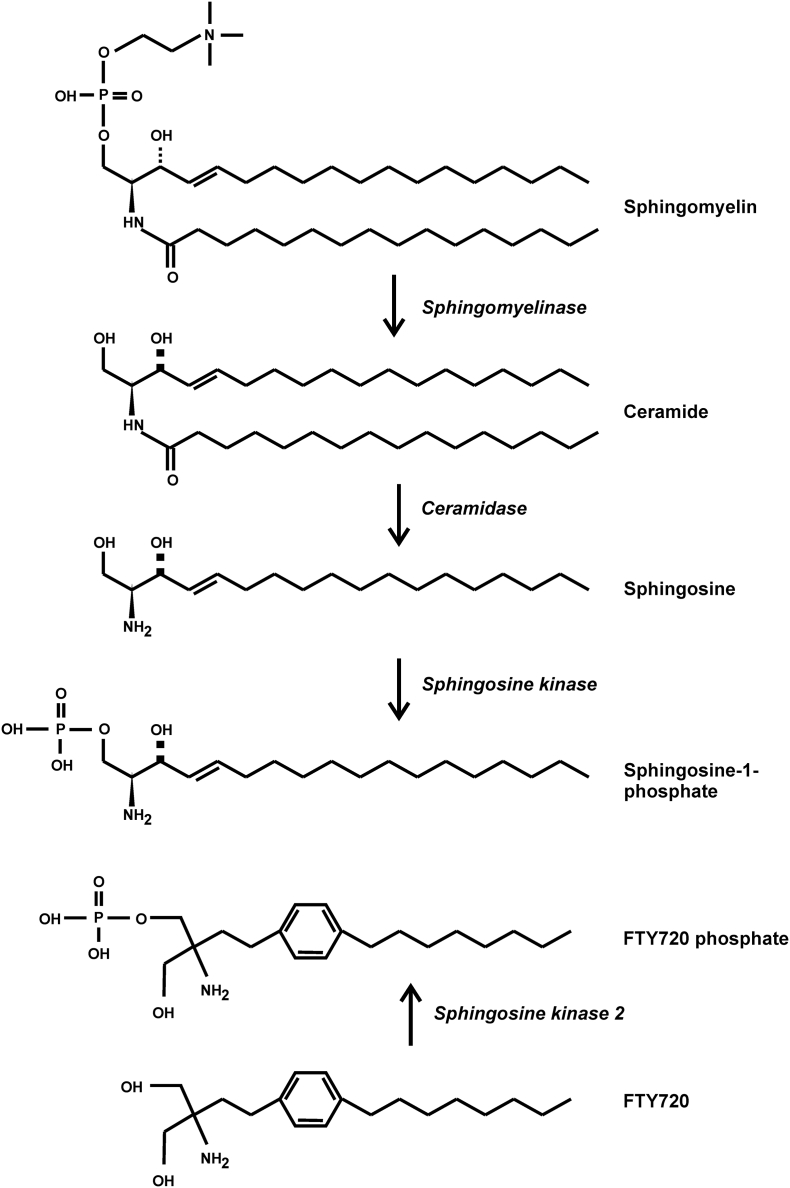
Sphingolipid metabolism. Representative structures of lipid compounds are illustrated. For comparison, structures of FTY-720 and phospho-FTY-720 are also shown.

Sphingolipids are pleiotropic lipid second messengers, which modulate cellular functions by several mechanisms (Aoki, Aoki, Ramanathan, Hait, & Takabe, 2016; Kunkel, Maceyka, Milstien, & Spiegel, 2013; Oaks & Ogretmen, 2014; Spiegel & Milstien, 2011). The polar, membrane associated sphingolipid sphingomyelin is cleaved by sphingomyelinases, releasing phosphocholine and ceramide (Fig. 2). Ceramide causes activation of PP2A (Chalfant, Szulc, Roddy, Bielawska, & Hannun, 2004; Cornell et al., 2009; Dobrowsky, Kamibayashi, Mumby, & Hannun, 1993; He, Du, Ke, Wen, & Zhang, 2019; Mukhopadhyay et al., 2009; Ruvolo, Deng, Ito, Carr, & May, 1999), an effect that is attributed to binding of the lipid to SET, and disruption of the inhibitory SET-PPP2CA interaction (Mukhopadhyay et al., 2009; Saddoughi et al., 2013). Further processing of ceramide has additional cell signaling consequences, which will be briefly discussed here because of their relevance to PP2A as a therapeutic target. Ceramidase enzymes cleave the acyl side chain from ceramide to yield sphingosine. This lipid is phosphorylated by sphingosine kinases 1 or 2 (Sphk1 and Sphk2) to generate sphingosine-1-phosphate (S1P), a lipid messenger with multiple, complex and context-dependent effects on the immune system (reviewed in Aoki et al., 2016; Kunkel et al., 2013; Maceyka, Harikumar, Milstien, & Spiegel, 2012; Spiegel & Milstien, 2011; Strub, Maceyka, Hait, Milstien, & Spiegel, 2010). Within the cell, S1P functions as a cofactor for TRAF2 (TNF receptor associated factor 2), an E3 ubiquitin ligase that plays an essential role in signaling from the TNF receptor to the transcription factor NF-κB (nuclear factor κ light chain enhancer of activated B cells) (Alvarez et al., 2010; Park et al., 2015; Spiegel & Milstien, 2011) (see below). It may also function in a similar fashion to facilitate Toll-like receptor signaling by enhancing the E3 ubiquitin ligase activity of TRAF6 (Spiegel & Milstien, 2011). Sustained activation of NF-κB by S1P contributes to enhanced expression of pro-inflammatory and pro-survival genes in the context of colitis-associated cancer (Liang et al., 2013). Other intracellular effects of S1P have been reported, including the inhibition of histone deacetylases 1 and 2 in the nucleus, overcoming the suppression of transcription by these epigenetic regulators (Ebenezer, Fu, Suryadevara, Zhao, & Natarajan, 2017; Hait et al., 2009; Yan et al., 2018). The gene specificity of transcriptional regulation by this mechanism is not fully understood.

The most well studied actions of S1P follow its export from the cell, principally by the transporter Spinster 2. Thereafter, S1P acts as an extracellular ligand for its receptors, S1PR1-5, in a process known as inside-out signaling. The five S1P receptors belong to the superfamily of G protein coupled receptors. The nature of the signal transduced by each S1P receptor is determined by the GTPase complexes with which it is associated at the cell membrane. This flexibility allows S1P to exert cell type- and context-specific effects. The engagement of S1PR2 or 3 on vascular endothelial cells promotes activation of NF-κB via coupling to G_α12/13_, the small GTPase protein RhoA and RhoA-activated kinase (Fernandez-Pisonero et al., 2012; Keul et al., 2011; Sanchez et al., 2007; Skoura et al., 2007; Zhang, Yang, et al., 2013; Zhang, An et al., 2013). This mechanism increases expression of adhesion molecules and inflammatory mediators, whilst impairing endothelial barrier function. In contrast the engagement of S1PR1 on vascular endothelial cells contributes to the maintenance of barrier function.

Another very important role of S1P-S1PR signaling is in the regulation of leukocyte traffic (Aoki et al., 2016; Spiegel & Milstien, 2011). Egress of leukocytes from lymphoid tissues is driven by an S1P gradient between lymphoid tissue, where concentration is low, to circulating blood or lymph, where high S1P concentrations are maintained by distinct mechanisms. The length of residency of leukocytes in lymphoid organs is regulated in part by adjusting the cell surface expression of the receptor S1PR1, making them more or less sensitive to the S1P gradient. The S1P analogue FTY720 (otherwise known as Fingolimod or Gilenya) is licensed for use in multiple sclerosis (MS), an auto-immune disease in which demyelination of neurons causes impairment of function, with consequent physical, cognitive and psychiatric symptoms (Brinkmann et al., 2010; Chaudhry, Cohen, & Conway, 2017; Mehling, Kappos, & Derfuss, 2011). FTY720 is phosphorylated by Sphk2 (Kharel et al., 2005; Paugh, Payne, Barbour, Milstien, & Spiegel, 2003; Zemann et al., 2006), and the product of this reaction causes internalization and proteasome-mediated degradation of S1P receptors (Graler & Goetzl, 2004; Mullershausen et al., 2009; Oo et al., 2007; Sykes et al., 2014). The consequent impairment of egress of lymphocytes from lymphoid tissues is thought to underlie therapeutic effects of FTY720 in MS (Brinkmann et al., 2010).

However, the effects of FTY720 are considerably more complex (Pitman, Woodcock, Lopez, & Pitson, 2012). FTY720-P initially functions as a potent agonist of S1P receptors, in particular S1PR1 (Brinkmann et al., 2002). Even when this receptor is internalized, it may continue to generate signals that influence cell behavior (Mullershausen et al., 2009). Consequently, there is often ambiguity as to whether effects of FTY720 *in vivo* are mediated by agonistic or antagonistic effects on S1P receptors. More relevant here, being structurally related to ceramide (Fig. 2), FTY720 also binds to SET, interrupting the interaction of the inhibitor with PP2A and promoting PP2A activation (Liu et al., 2008; Matsuoka, Nagahara, Ikekita, & Shinomiya, 2003; Neviani et al., 2007; Neviani et al., 2013; Pippa et al., 2014; Roberts et al., 2010; Saddoughi et al., 2013; Yang, Huang, Lu, Li, & Huang, 2012). Throughout this review, we will return to this intriguing drug and its derivatives, their mechanisms of action and actual or potential clinical uses.

## 3 PP2A and cancer

### 3.1 Dysregulation of PP2A in cancer

As reviewed in depth elsewhere, impairment of PP2A function is a very common feature of many cancers, therefore PP2A may be considered as a tumor suppressor (Carratu, Signorile, De Rasmo, Reale, & Vacca, 2016; Grech et al., 2016; Haesen et al., 2014; Janssens & Rebollo, 2012; O'Connor, Perl, Leonard, Sangodkar, & Narla, 2017; Ruvolo, 2016; Westermarck & Hahn, 2008). Dysregulated phosphorylation and function of PP2A substrates contributes to several hallmarks of cancer (Hanahan & Weinberg, 2011), including inflammation as discussed in detail below. In cancer, many different routes to functional impairment of PP2A have been described, reflecting the magnitude of the advantages that can be gained in terms of growth, survival and metastasis. (1) Okadaic acid, calyculin A and microcystin-LR are toxins derived from aquatic microorganisms, which exert tumor-promoting effects by directly inhibiting PP2A (Fujiki & Suganuma, 2009). These and related compounds can be useful for investigating functions of PP2A, although their effects must be interpreted with caution because of their imperfect specificity for PP2A (Swingle, Ni, & Honkanen, 2007). (2) Small and middle T antigens of DNA tumor viruses also directly inhibit PP2A function, promoting proliferation of infected cells and conferring a replicative advantage to the virus (Chen et al., 2007; Cho et al., 2007; Guergnon et al., 2011; Sablina & Hahn, 2008). (3) Oncogenically dysregulated tyrosine kinases impair PP2A function (Chen et al., 1994; Zonta et al., 2015), although the precise mechanism remains uncertain as noted above. (4) Expression of specific B subunits is decreased through chromosomal deletion, epigenetic or micro-RNA-mediated silencing (Ruvolo, 2015). (5) Somatic mutations within the scaffolding subunit PPP2R1A specifically influence recruitment of particular B subunits (Haesen et al., 2016; Ruediger, Ruiz, & Walter, 2011). (6) Expression of negative regulators such as CIP2A and SET/is elevated (Hung & Chen, 2017; Kauko & Westermarck, 2018; Soofiyani, Hejazi, & Baradaran, 2017). (7) Expression of positive regulators such as PPP2R4 is decreased (Sents et al., 2017). For additional references see the review articles mentioned at the start of this paragraph.

### 3.2 Therapeutic targeting of PP2A in cancer

As reviewed extensively elsewhere (Carratu et al., 2016; Grech et al., 2016; Lambrecht et al., 2013; O'Connor et al., 2017; Perrotti & Neviani, 2013), PP2A-activating drugs (PADs) are being sought and developed by cancer researchers in academia and the pharmaceutical industry. One approach is based on the observation that the licensed drug FTY720 binds to the endogenous inhibitor protein SET, causing derepression of PP2A activity (Liu, Zhao, et al., 2008; Matsuoka et al., 2003; Neviani et al., 2007; Neviani et al., 2013; Pippa et al., 2014; Roberts et al., 2010; Saddoughi et al., 2013; Yang et al., 2012). This suggested that the immunomodulatory drug might be repurposed as an anti-tumor agent (Enjeti, D'Crus, Melville, Verrills, & Rowlings, 2016; Patmanathan, Yap, Murray, & Paterson, 2015; Rincon et al., 2015). FTY720 itself displayed promising effects in several pre-clinical cancer models (Baldacchino et al., 2014; Cristobal et al., 2014; Cristobal et al., 2016; Garner et al., 2018; Neviani et al., 2007; Oaks et al., 2013; Ramaswamy, Spitzer, & Kentsis, 2015; Szymiczek et al., 2017; Velmurugan et al., 2018; Wallington-Beddoe et al., 2012; Wallington-Beddoe, Hewson, Bradstock, & Bendall, 2011; Zonta et al., 2015). In some cases it overcame resistance to kinase inhibitors and chemotherapeutics that are commonly used as first line cancer treatments (Kiyota et al., 2013; McDermott et al., 2014; Neviani et al., 2013; Perrotti & Neviani, 2006; Rincon et al., 2015; Smith et al., 2016), indicating that combination therapies incorporating FTY720 or other PADs may be highly promising (Mazhar, Taylor, Sangodkar, & Narla, 2019).

**Fig. 3:**
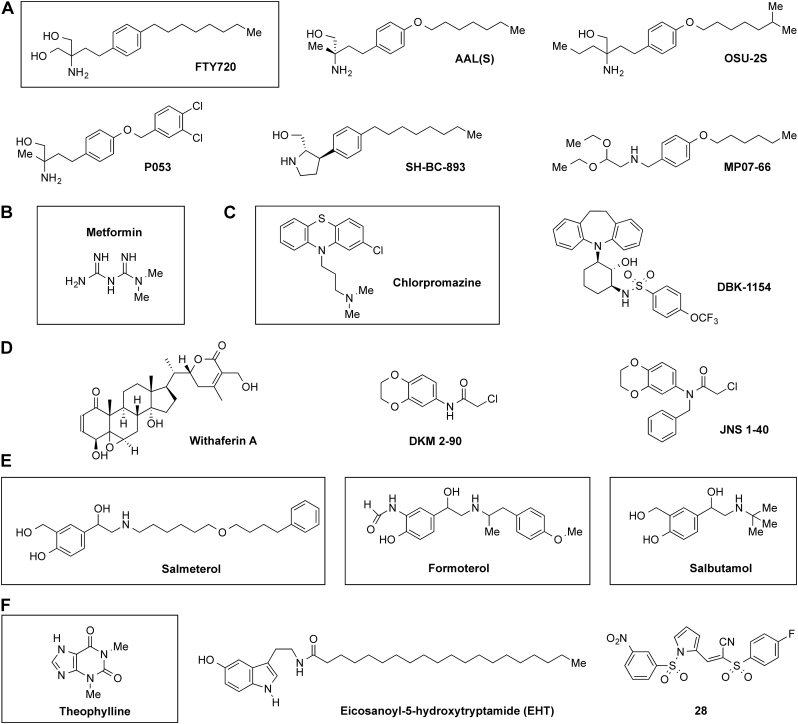
Therapeutic targeting of PP2A. Various compounds reported to activate PP2A are shown, with licensed drugs boxed. A) FTY720 and derived compounds; B) Metformin; C) the anti-psychotic phenothiazine chlorpromazine and daughter compound DBK-1154; D) compounds reported to promote alkylation of PPP2CA; E) β-adrenergic receptor agonists; F) caffeine-related compounds theophylline and EHT, and the PME-1 inhibitor compound 28.

Anti-tumor actions of FTY720 do not require its phosphorylation. In fact, lymphopenia and immuno-suppression driven by antagonism of S1P receptors are considered to be potential obstacles to the use of FTY720 in cancer. Therefore, some researchers have sought to identify FTY720 derivatives that cannot be phosphorylated, yet retain anti-tumor effects independent of the S1P-S1PR axis (Toop et al., 2016). FTY720 derivatives known as AAL(s) and OSU-2S were generated by substitution of the phosphorylatable pro-R hydroxyl group (Kiuchi et al., 1998; Omar et al., 2011) (Fig. 3A). These molecules cannot be phosphorylated or bind to S1PRs, but retain the SET-binding, PP2A-activating and anti-tumor properties of their parent (Mani et al., 2015; Mani et al., 2017; Omar et al., 2011; Omar, Tolba, Hung, & Al-Tel, 2016; Roberts et al., 2010; Smith et al., 2016). Replacement of the saturated lipophilic chain may also be possible while retaining PP2A-activating properties, as for example in P053 (Turner et al., 2018) (Fig. 3A). Edinger and colleagues have developed aza-cyclic FTY720 analogs that are again not phosphorylated but retain SET-binding and PP2A-activating properties (for example SH-BC-893; Fig 3A) (Kim et al., 2016; Kubiniok et al., 2019; McCracken et al., 2017). They are claimed to have reduced cardio-vascular and other known off-target effects of FTY-720 (Chen et al., 2016; Perryman et al., 2016). A third line of medicinal chemistry led to SET-binding compounds exemplified by MP07-66 (Tibaldi et al., 2017; Zonta et al., 2015) (Fig. 3A). Although these compounds generally retain the amphiphilic, sphingolipid-like structure of FTY-720, no assumptions can be made about their off-target effects and safety profiles, and little information is yet available. Apolipoprotein E-derived peptides such as COG1410 also bound to SET, activated PP2A (Christensen et al., 2011) and exerted anti-tumor effects in several experimental models of cancer (Agarwal et al., 2014; Christensen et al., 2011; Fujiwara et al., 2013; Hu et al., 2015; Richard et al., 2016; Shlomai et al., 2017; Switzer et al., 2011). The precise mechanism of action and pathway to translation of these peptides are unclear. The utility of all of these diverse SET-binding molecules depends upon how strongly PP2A activity is regulated by SET in the relevant cellular context.

The biguanidine compound metformin (Fig. 3B), widely used as an anti-diabetic drug, was reported to interrupt the interaction between the catalytic subunit PPP2CA, the regulator of PP2A holoenzyme assembly IGBP1, and the ubiquitin E3 ligase Midline 1 (Demir, Koehler, Schneider, Schweiger, & Klocker, 2014; Kawashima & Kirito, 2016; Kickstein et al., 2010; Zhang et al., 2019). It is thought that metformin releases PP2CA and IGBP1 from an inhibitory effect of Midline 1, promoting the assembly of functional PP2A holoenzyme complexes. Further work is required to clarify the mechanism of action of metformin on PP2A, and the extent to which this contributes to anti-inflammatory effects of metformin (Saisho, 2015).

Other PP2A activating compounds interact directly with components of the PP2A holoenzyme. Anti-psychotic phenothiazine drugs such as chlorpromazine (Fig. 3C) have anti-proliferative effects, but could not be used to treat cancer because of harmful side-effects related to engagement of G protein-coupled receptors (GPCRs) and amine transporters. A key mechanistic finding was that phenothiazines promoted PP2A-mediated nuclear localization of the forkhead box transcription factor FOXO1, leading to inhibition of cell division (Gutierrez et al., 2014; Kau et al., 2003; Yan et al., 2008). In parallel it was discovered that effects mediated by GPCRs and amine transporters could be eliminated while retaining PP2A-mediated anti-proliferative properties (Kastrinsky et al., 2015). This led to tricyclic sulfonamide compounds such as DBK-1154 (Fig. 3C), which bind in the HEAT repeats of the PP2A scaffold protein PPP2R1A at a site proximal to the C-terminal of the catalytic subunit, eliciting robust PP2A activation and impairing growth of non small cell lung cancer, prostate and pancreatic cancers (Allen-Petersen et al., 2019; Kauko et al., 2018; McClinch et al., 2018; Sangodkar et al., 2017; Tohme et al., 2019). *In vitro*, anti-proliferative effects on non small cell lung cancer cells were accompanied by extensive changes to the phospho-proteome, consistent with PP2A-mediated inactivation of pathways that promote cell cycle progression (Wiredja et al., 2017). Grossman and colleagues have reported that the complex plant-derived lactone withaferin A (Fig. 3D) alkylates a specific cysteine residue of PPP2R1A and elicits an irreversible PP2A activating effect (Grossman et al., 2017). Further medicinal chemistry development led to a series of simpler, synthetic molecules that promoted alkylation of the same PPP2R1A cysteine residue. These are exemplified by DKM 2-90 and JNK 1-40 (Fig. 3D). Compounds that directly target PP2A might be expected to have broader activity than those which target one specific PP2A inhibitor. It remains to be seen whether this translates to a clinical advantage, whether reversible or irreversible PP2A activation is preferable, or whether alkylating agents have utility outside of cancer therapy.

Various other molecules have been reported to activate PP2A with potentially therapeutic effect. Amongst these are salmeterol, formoterol and salbutamol (Fig. 3E), agonists of β-adrenergic receptors that are commonly used in the treatment of asthma (Bastan, Eskandari, Ardakani, & Peachell, 2017; Hatchwell et al., 2014; Kobayashi, Mercado, Miller-Larsson, Barnes, & Ito, 2012; Sokulsky et al., 2016); theophylline (Fig. 3F), a methylxanythine drug closely related to caffeine, and used to treat asthma and chronic obstructive pulmonary disease (Patel et al., 2016); and eicosanoyl-5-hydroxytryptamide (EHT; Fig. 3F), a related compound that was discovered in coffee and is now marketed as a nutraceutical product (K. W. Lee, Im et al., 2013). EHT may influence PP2A activity by modulating the methylation state of the C-terminal leucine residue. Other researchers have sought to optimize specific inhibitors of the demethylase enzyme PME-1 (Bachovchin et al., 2011; Zuhl et al., 2012), leading to the discovery of sulfonyl acrylonitriles exemplified by Compound 28 (Fig. 3F). These compounds are described as experimental tools, yet may have some anti-cancer applications (Kaur & Westermarck, 2016; Pusey et al., 2016).

As highlighted in Fig. 3, molecules of several distinct chemical classes have been shown to activate PP2A in different ways, targeting the PP2A holoenzyme itself or its regulators by reversible and irreversible mechanisms. Differences of composition and mechanism of action are very likely to result in differences of both on- and off-target cellular effects, context-dependent efficacy, toxicity and tolerability. It is worth pointing out that several licensed drugs with well-known and broadly acceptable safety profiles have been reported to activate PP2A by direct, indirect or as yet unknown mechanisms. Arguably this increases the likelihood that drugs which are actually designed to activate PP2A will be tolerated.

## 4 Involvement of PP2A in the control of inflammation

### 4.1 Introduction

**Fig. 4:**
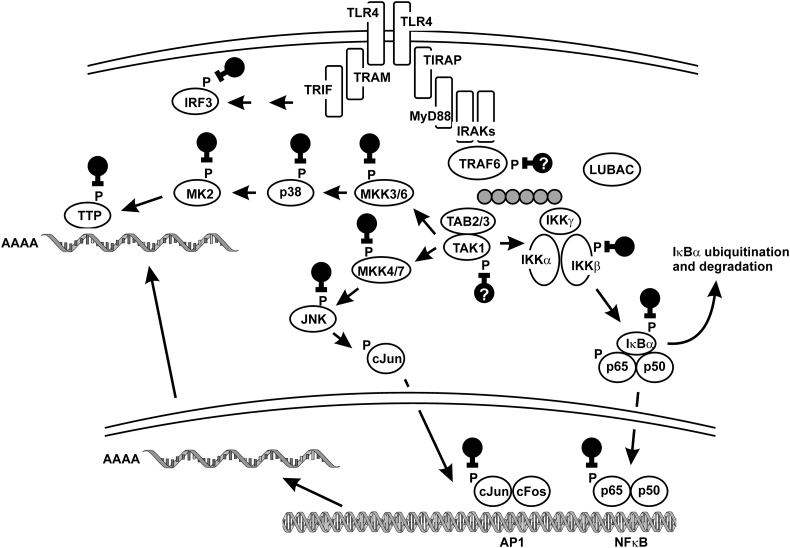
Sites of action of PP2A in Toll-like receptor 4- (TLR4-) mediated signaling. TLR4 regulates gene expression via the assembly of large signaling complexes and the activation of various phosphorylation-mediated signaling cascades, which are shown here in cartoon form. Signals via TRAM and TRIF to IRF3 originate from an endosomal compartment, which for reasons of simplicity is not indicated here. Black symbols represent protein dephosphorylation by PP2A. Grey circles represent linear or K63-linked poly-ubiquitin chains. AP-1, activator protein 1; IκBα, α inhibitor of NF-κB; IKK, IκBα kinase; IRF, interferon-regulatory factor; JNK, cJun N-terminal kinase; LUBAC, linear ubiquitin chain assembly complex; MAPK, mitogen-activated protein kinase; MK2, MAPK-activated kinase 2; MKK, MAPK kinase; MyD88, myeloid differentiation factor 88; NF-κB, nuclear factor κ enhancer of activated B cells; TAB, TAK1 binding protein; TAK1, transforming growth factor β-activated kinase; TIRAP, Toll/interleukin 1 receptor domain-containing adaptor protein; TLR, Toll-like receptor; TRAF, TNF receptor interacting factor; TRAM, Toll/interleukin 1 receptor domain-containing adaptor molecule; TRIF, Toll/interleukin 1 receptor domain-containing adaptor inducing interferon β; TTP, tristetraprolin.

The interactions between pathogen-associated molecular patterns (PAMPs) and their cognate pattern recognition receptors (PRRs) are the bedrock of innate immunity and inflammation (O'Neill, Golenbock, & Bowie, 2013). Toll-like receptor 4 (TLR4), the first PRR to be identified, is expressed on the surface of many cell types, but most notably monocytes, macrophages and dendritic cells. With the assistance of accessory proteins, TLR4 recognizes LPS, an essential component of the cell wall of gram negative bacteria. Upon binding of LPS, TLR4 triggers a signaling cascade that promotes expression of cytokines, chemokines, other inflammatory mediators and microbicidal gene products (Arthur & Ley, 2013; O'Neill et al., 2013). PAMPs recognized by other PRRs are commonly structural components of pathogens, or forms of nucleic acid that are associated with pathogens. These are interpreted by the cell as generic evidence of the presence of microbes, and all are capable of triggering inflammatory responses. There are subtle differences in the signaling pathways engaged and the programmes of gene expression activated, allowing cells to tailor their responses to the nature of the challenge. Pro-inflammatory cytokines such as interleukin 1β (IL-1β) and tumor necrosis factor α (TNFα) bind to their own cell surface receptors, and the initial stages of their signaling pathways differ from those of the PRRs. However, common signaling components are engaged downstream, and overlapping phosphorylation-dependent mechanisms are deployed to bring about changes of cellular function. Here we focus on three signaling pathways in which there is strong evidence for involvement of PP2A (Fig. 4).

### 4.2 NF-κB (nuclear factor κ light chain enhancer of activated B cells)

The binding of LPS to TLR4 induces a conformational change in the intracellular domain of the receptor, which drives the assembly of a very high molecular weight signaling complex, culminating in the recruitment and activation of tumor necrosis factor associated factor (TRAF6; Fig. 4). TRAF6 is an E3 ubiquitin ligase, which catalyses the formation of long chains of covalently linked ubiquitin peptides attached either to TRAF6 itself or to other components of the signaling complex. First, an isopeptide bond is created between the C-terminal glycine residue of a single, 76 amino acid ubiquitin polypeptide and a lysine residue of the acceptor protein. Then the chain is extended by sequential addition of several more ubiquitin polypeptides, each forming an isopeptide bond to lysine 63 of the preceding ubiquitin moiety. In other words these ubiquitin chains are “K63-linked”. Chains created by the linear ubiquitin assembly complex (LUBAC) are also increasingly recognized as important contributors to inflammatory responses (Shimizu, Taraborrelli, & Walczak, 2015). In either case, poly-ubiquitin chains create scaffolds for recruitment and activation of other signaling molecules, which will ultimately convert the TLR4 signal into transcriptionally- and post-transcriptionally-mediated changes in the expression of inflammatory and antimicrobial genes. One of the most important mediators of this response is the transcription factor NF-κB (Taniguchi & Karin, 2018). In resting cells, NF-κB is cytoplasmically sequestered in an inactive form through masking of its nuclear localisation signal by IκBα (inhibitor of NF-κB α). The activation of NF-κB requires degradation of IκBα, which is accomplished as follows. Transforming growth factor β (TGFβ)-associated kinase 1 (TAK-1) is recruited to the TLR4 signaling complex via ubiquitin binding domains of the associated TAK-1-binding proteins 2 or 3 (TAB-2/3). The IκBa kinase (IKK) complex is recruited via a ubiquitin-binding domain of the scaffold protein IKKγ. TAK-1 molecules, brought into proximity via clustering at the poly-ubiquitin scaffold, activate one another via phosphorylation of threonines 184 and 187. They then activate IKKβ via phosphorylation of serines 177 and 181. Once activated, IKKβ phosphorylates IκBα at serines 32 and 36. These phosphorylations promote poly-ubiquitination of IκBα with K48 linkages (ie each new ubiquitin molecule is ligated to lysine 48 of the preceding molecule). Unlike the K63 chain, this post-translational modification is recognized as a signal for protein degradation by the proteasome. As a consequence of IκBα degradation, NF-κB is released from its repressive complex and allowed to migrate to the nucleus, where it activates transcription of large numbers of pro-inflammatory, anti-microbial and anti-apoptotic genes. The process is further modulated by additional phosphorylation events (Christian, Smith, & Carmody, 2016; Huang, Yang, Lamb, & Chen, 2010; Neumann & Naumann, 2007). RelA (reticuloendotheliosis viral oncogene homolog A), a component of the canonical NF-κB dimer, is phosphorylated by IKKβ at Ser536, a modification associated with transcriptional activation. A variety of other kinases have been reported to modulate transcription via the phosphorylation of RelA or other NF-κB components (Christian et al., 2016; Huang et al., 2010; Neumann & Naumann, 2007).

The NF-κB signaling pathway depends on serine-threonine phosphorylation at several points, suggesting that it may be regulated by serine-threonine phosphatases. Okadaic acid, calyculin A and microcystin-LR are all reported to promote nuclear localization, phosphorylation and transcriptional activation of NF-κB in a wide variety of cell types (Feng, Ohmori, & Chang, 2006; Grossman, Shanley, Denenberg, Zhao, & Wong, 2002; Miskolci et al., 2003; Ozaki et al., 2006; Tanaka et al., 2007; Thevenin et al., 1990; Zhang et al., 2012). In many cases the PP2A inhibitors, alone or in combination with agonists such as LPS, TNFα or IL-1β, increased the expression of known NF-κB target genes, including pro-inflammatory mediators or regulators of apoptosis. As remarked above, such observations need to be interpreted with caution because of the imperfect specificity of the compounds as PP2A inhibitors (Swingle et al., 2007). However, there is a wealth of corroborating evidence. Expression of SV40 small T antigen also activated NF-κB (Moreno et al., 2004; E. Sontag, Sontag, & Garcia, 1997). PP2A physically interacts with the IKK complex, IκBα and RelA itself and can catalyse the removal of phosphates that are required for the activation or proteolytic processing of these proteins (DiDonato, Hayakawa, Rothwarf, Zandi, & Karin, 1997; Li, Wang, Berman, Zhang, & Dorf, 2006; Tsuchiya et al., 2017; J. Yang, Fan, Wadzinski, Sakurai, & Richmond, 2001). Macrophage-specific disruption of the mouse *Ppp2ca* gene enhanced the expression of several inflammatory mediators in LPS-injected mice, and strongly increased mortality in response to this challenge (He et al., 2019; L. Sun et al., 2017). LPS-stimulated *Ppp2ca-/-* macrophages displayed noticeable prolongation of IKKβ phosphorylation, and more dramatic prolongation of RelA Ser536 phosphorylation. The phosphorylation of IKKα/β was increased by knock-down of PPP2CB in astrocytes (Li et al., 2006) or of PPP2CA in KB cells (Witt et al., 2009). These observations powerfully illustrate the important role of PP2A in the limitation of inflammatory responses, but do not yet shed light on mechanisms or specificity of targeting of the NF-κB pathway by PP2A at different levels.

A systematic RNA interference (RNAi) screen was used to identify phosphatase components that regulate the NF-κB pathway in a mouse astrocyte cell line. NF-κB activity was enhanced by knock-down of the B subunit PPP2R5C (as well as by knock-down of scaffolding and catalytic subunits) (Li et al., 2006). PPP2R5C was reported to mediate dephosphorylation of a novel activatory phosphorylation site in TRAF2, Thr117 (Li, Wang, & Dorf, 2009). Knock-down of PPP2R5C also decreased IκBα levels in human embryonic kidney cells, consistent with activation of NF-κB signaling (Moreno et al., 2004). In another RNAi screen (Breuer et al., 2014), knock-down of PPP2R5C in a T cell line enhanced the activation of an NF-κB reporter construct by TNF, 12-O-tetradecanoylphorbol-13-acetate or T cell receptor engagement, whereas over-expression of the same B subunit decreased NF-κB activity and the expression of IL-2. TCR engagement increased the expression of PPP2R5C, presumably as a negative feedback mechanism limiting the duration of the NF-κB activation response. Knock-down of PPP2R5C increased the phosphorylation of IKKα/β, suggesting that the site of action is at this level or above. Mathematical modeling of the NF-κB signaling pathway implied that PP2A constitutively associates with and negatively regulates the IKK complex in KB cells (Witt et al., 2009). However, in dendritic cells *de novo* gene expression was required for PP2A to interact with the IKK complex and promote IKKα/β dephosphorylation (Chang, Voorhees, Liu, Zhao, & Chang, 2010). One obvious interpretation is that LPS induces the expression of a B subunit that is essential for targeting of PP2A to IKK and terminating the activation of the NF-κB pathway. However, the systematic RNAi screens described above did not conclusively identify an IKK targeting factor (Breuer et al., 2014; Li et al., 2006). An over-expression approach in HEK293 (a human kidney cancer cell line) implicated members of the STRN family as possible IKK targeting B subunits (Tsuchiya et al., 2017). This awaits corroboration by knock-out or knock-down methods.

NF-κB-dependent genes are differentially affected by variations in the dynamics of NF-κB activation (Brasier, 2006), providing a mechanism by which PP2A can exert gene-specific effects. For example in dendritic cells PP2A prevents sustained activation of the IKK complex and NF-κB, inhibiting the expression of *Il23b* without influencing expression of other members of the IL-12 family (Chang et al., 2010). In contrast, the interaction between PP2A and the IKK complex is targeted by the human T-lymphotropic virus 1 transcriptional activation protein Tax, promoting sustained IKK activation and NF-κB mediated transcription from the viral long terminal repeat (Fu, Kuo, Liu, Jeang, & Giam, 2003; Hong et al., 2007). Ultraviolet B radiation (UVB) impairs PP2A-mediated dephosphorylation and inactivation of IKKα/β, prolonging NF-κB activation. This contributes to cooperative regulation of TNF and other NF-κB target genes by IL-1 and UVB (Barisic, Strozyk, Peters, Walczak, & Kulms, 2008; Witt et al., 2009). The mechanism of inactivation of PP2A by UVB is not known, but is thought to be indirect (Witt et al., 2009).

There is increasing evidence that PP2A also directly targets RelA to control the duration of NF-κB activation. First, PP2A interacts with RelA and promotes its dephosphorylation (Hsieh, Hsiao, Hsu, Wang, & Sheu, 2014; Yang et al., 2001). Plant-derived anti-inflammatory compounds have been shown to decrease the phosphorylation of RelA at Ser576, apparently without influencing upstream signaling events (Hsieh et al., 2011; Hsieh et al., 2014; Shu et al., 2016). Although the basis of targeting of RelA by PP2A is not yet known, an endogenous factor that influences this process has been identified (Zhang, Park, et al., 2013). Pleckstrin homology domain finger protein 20 recognizes methylated Lys221 of RelA, protecting RelA against PP2A-mediated dephosphorylation of Ser576, and thereby prolonging NF-κB activity in the nucleus.

PP2A may exert subtle cell type-, gene- and stimulus-specific effects on gene expression by regulating inflammatory signaling at several levels, modulating the dynamics of NF-κB activation and inactivation rather than imposing a simple on/off switch. PP2A dephosphorylates and inactivates TAK1 to negatively regulate TGFβ1 signaling in mesangial cells (Kim, Kwak, Wang, & Choi, 2008), but has not yet been shown to regulate NF-κB activation via TAK1. Identification of targeting mechanims in the NF-κB pathway remains challenging, perhaps because of redundancy between B subunits, or because important mediators of PP2A substrate specificity have yet to be identified. Finally, impaired PP2A function and dysregulated NF-κB activity may contribute to pathological conditions such as viral replication and oncogenic transformation (Guergnon et al., 2011; Taniguchi & Karin, 2018). It is also worth noting that myeloid-specific disruption of the *Ppp2ca* gene resulted in over-expression of Interferon β and interferon-regulated genes, which was attributed to enhanced function of NF-κB, a known transcriptional regulator of the *Ifnb1* gene (Sun et al., 2017). There may be an involvement of the transcription factor IRF3, which also promotes *Ifnb1* transcription, and is activated via phosphorylation downstream of recruitment of the adaptor protein TRIF to TLR4 and other PRRs at the endosomal compartment. PP2A was shown to mediate dephosphorylation and inactivation of IRF3, with the involvement of two unexpected adapter proteins, receptor for activated C kinase 1 (Long et al., 2014) and F-box protein 17 (D. Peng, Wang, Huang, Zhao, & Qin, 2017).

### 4.3 MAPKs (mitogen-activated protein kinases)

Downstream of TLR4 engagement, TAK-1 directly or indirectly mediates the activation of various mitogen-activated protein kinase kinases (MKKs) (Arthur & Ley, 2013; Pattison et al., 2016). In turn, these dual specificity kinases phosphorylate their substrate MAPKs at closely spaced threonine and tyrosine residues, causing activation. Of the three classical MAPK families, p38 and JNK (cJun N-terminal kinase) are intimately linked to the regulation of inflammatory gene expression, whereas ERK (extracellular signal-regulated kinase) appears to play a less prominent role in inflammatory responses (Arthur & Ley, 2013). MAPK p38 controls expression of inflammatory genes via the phosphorylation of transcription factors and via the regulation of mRNA stability (see below). As its name implies, JNK is largely responsible for the activation of c-Jun via phosphorylation of serines 63 and 73 within its N-terminal transcriptional activation domain. As one component of the dimeric transcription factor AP-1 (activator protein 1), c-Jun contributes to the transcriptional activation of large numbers of pro-inflammatory mediators, often functioning collaboratively with transcription factors of the ETS (E twenty-six), IRF (interferon-regulatory factor) and NF-κB families (Natoli, Ghisletti, & Barozzi, 2011; Peng, 2008; Wagner, 2010). Aberrant activation of the JNK pathway has been implicated in pathogenesis of several diseases, including some inflammation-associated cancers and meta-inflammation associated with insulin resistance (Sabapathy, 2012).

The activation of MAPKs in response to pro-inflammatory stimuli is enhanced and/or prolonged when PP2A function is compromised by pharmacological inhibition, knock-down or knock-out of catalytic subunits, or overexpression of inhibitors. This leads to enhanced expression of inflammatory mediators including cytokines, chemokines and metalloproteinases (Al-Murrani, Woodgett, & Damuni, 1999; He et al., 2019; Kamat, Rai, Swarnkar, Shukla, & Nath, 2014; Law, Tam, Lee, & Lau, 2013; Rahman et al., 2015; Shanley, Vasi, Denenberg, & Wong, 2001; Sun et al., 2017; Wang et al., 2017; Westermarck, Holmstrom, Ahonen, Eriksson, & Kahari, 1998). In several cases, an increase in JNK-mediated activation of AP-1 has been implicated in the augmented inflammatory responses. Consistent with this proposed mechanism, PP2A interacts with JNK and its upstream activator MKK4 (Avdi, Malcolm, Nick, & Worthen, 2002; Zhao et al., 2008). As an added complication, nuclear PP2A complexes containing the B subunit PPP2R2A can reverse the phosphorylation of cJun at threonine 239, a post-translational modification that impairs transcriptional activation (Gilan et al., 2015; Shi et al., 2017). Hence PP2A may either positively or negatively regulate AP-1 function, depending on the expression of B subunits and their subcellular localization. Yet another level of complexity is introduced by PP2A-mediated cross-talk between MAPK signaling pathways. MAPK p38-dependent increase of PP2A activity causes a decline in activity of JNK or ERK (Avdi et al., 2002; Junttila, Li, & Westermarck, 2008; Liu, Chen, Cheng, Lin, & Chang, 2013; Liu & Hofmann, 2004; Wang et al., 2006; Westermarck, Li, Kallunki, Han, & Kahari, 2001). Such cross-talk is likely to involve phosphorylation-mediated modulation of specific PP2A holoenzyme complexes, but the molecular mechanisms are not yet known.

### 4.4 TTP (Tristetraprolin)

MAPK p38 phosphorylates and activates the downstream kinase MK2 (MAPK-activated protein kinase 2), which has multiple roles in the regulation of cellular responses to stress and pro-inflammatory stimuli (Gaestel, 2013; Menon & Gaestel, 2018). One important downstream target is tristetraprolin (TTP), a member of a small family of zinc finger mRNA-binding proteins, encoded by the gene *Zfp36* (mouse) or *ZFP36* (human) (Brooks & Blackshear, 2013). TTP binds in a sequence-specific manner to sites closely resembling the sequence [A/U]UAUUUAU[A/U]. It then recruits several nuclease proteins or protein complexes, amongst which the most important is the CCR4/NOT deadenylase complex. This catalyses the progressive removal of the protective poly-(A) tail, the rate-limiting step in the degradation of most mRNA species (Carpenter, Ricci, Mercier, Moore, & Fitzgerald, 2014; Newman, McHugh, & Turner, 2016). Cognate binding sites for TTP are commonly found in the 3’ untranslated regions of mRNAs that encode pro-inflammatory mediators, as well as regulators of cell cycle progression. Therefore, just as NF-κB is a master transcriptional regulator of inflammatory responses, TTP can be considered as a master negative regulator of inflammatory responses at the post-transcriptional level (Brooks & Blackshear, 2013). Germline disruption of the *Zfp36* gene causes a severe inflammatory syndrome characterized by increased stability of many pro-inflammatory transcripts and over-expression of their products, most notably tumor necrosis factor α (TNFα) (Carballo, Gilkeson, & Blackshear, 1997; Carballo, Lai, & Blackshear, 1998; Taylor et al., 1996).

TTP is very extensively post-translationally modified, with at least thirty well-documented sites of phosphorylation (Cao et al., 2006; Clark & Dean, 2016; O'Neil, Ammit, & Clark, 2018; Sandler & Stoecklin, 2008). Little or nothing is known about the mechanisms and consequences of most of these phosphorylations. However, MK2 is known to mediate the phosphorylation of three sites, namely Ser52, Ser178 and Ser316 (Chrestensen et al., 2004; Hitti et al., 2006; Stoecklin et al., 2004). Ser316 is within a highly conserved C-terminal domain that is critical for regulation of mRNA stability by TTP and its close relatives (Blackshear & Perera, 2014). The phosphorylation of this site is thought to impair the mRNA-destabilizing function of TTP, although the mechanism has not yet been fully characterized (Fabian et al., 2013). The phosphorylation of Ser52 and Ser178 facilitates binding of TTP by 14-3-3 proteins (Chrestensen et al., 2004), adaptors that specifically recognise the phosphorylated forms of their client proteins (Mackintosh, 2004; Wilker & Yaffe, 2004). The recruitment of 14-3-3 proteins impairs the interaction of TTP with the CCR4/NOT complex, thus promoting stabilization of mRNAs that would otherwise be targeted for rapid degradation (Marchese et al., 2010; Sandler, Kreth, Timmers, & Stoecklin, 2011; Stoecklin et al., 2004; Sun et al., 2007).

Because it lacks defined structure outside of its central RNA-binding domain, TTP protein tends to be rapidly turned over by a proteasome-mediated but ubiquitination-independent unfolded protein degradation pathway (Ngoc et al., 2014). A second consequence of the phosphorylation of Ser52 and Ser178 is protection of TTP against this process (Brook et al., 2006; Hitti et al., 2006; Ngoc et al., 2014). 14-3-3 proteins are known to impose stable structure upon their phosphorylated client proteins (Yaffe, 2002). It remains to be demonstrated that 14-3-3 protein recruitment mediates the stabilization of TTP in response to its phosphorylation (Clark & Dean, 2016; Ngoc et al., 2014). In any case, the result of the coupled, phosphorylation-mediated stabilization and inactivation of TTP protein is that there is often an inverse relationship between the amount of TTP present and its activity. For example, in cells where the MAPK p38 pathway is dysregulated, phosphorylated TTP accumulates and TTP-regulated pro-inflammatory mRNAs are abnormally stable, resulting in exaggerated inflammatory responses (Abraham & Clark, 2006; Clark & Dean, 2016; Smallie et al., 2015). Similarly, okadaic acid treatment of airway epithelial cells increased the expression of the TTP targets IL-6 and IL-8, despite also increasing the expression of TTP protein (presumably in its phosphorylated and inactive form) (Rahman et al., 2015). Conversely, if TTP phosphorylation is blocked by targeted germline mutation of codons 52 and 178 of the mouse *Zfp36* gene, TTP protein is weakly expressed but highly active (Ross et al., 2015). The mutant mice (known as *Zfp36aa/aa*) are significantly protected in several experimental models of inflammatory pathology, including LPS-induced endotoxemia (O'Neil et al., 2017; Ross et al., 2015; Tang et al., 2017), zymosan-induced air pouch inflammation, experimental arthritis (Ross et al., 2017), cigarette smoke-induced airway inflammation and polymicrobial sepsis (unpublished observations).

The unusual relationship between quantity and activity of TTP may help to explain the otherwise puzzling observation, that TTP protein is abundant at sites of chronic inflammation such as the rheumatoid synovium or atherosclerotic plaque (Brooks, Connolly, Diegel, Fava, & Rigby, 2002; Ross et al., 2017; Zhang, Taylor et al., 2013). We have hypothesized that accumulation of phosphorylated, inactive TTP contributes to the establishment of chronic inflammation *in vivo* (Clark & Dean, 2016; Ross et al., 2017). TTP has also been identified as a putative tumor suppressor (Park, Lee, & Kang, 2018; Ross, Brennan-Laun, & Wilson, 2012; Sanduja, Blanco, Young, Kaza, & Dixon, 2012). It negatively regulates several genes that are involved in tumor progression, including cMyc (Rounbehler et al., 2012), a transcription factor that coordinates metabolic reprogramming of cancer cells (Carroll, Freie, Mathsyaraja, & Eisenman, 2018; Dejure & Eilers, 2017); and programmed death ligand 1 (Coelho et al., 2017), a cell surface molecule that aids immune evasion by tumors (Blank, Gajewski, & Mackensen, 2005; Dong, Sun, & Zhang, 2017; Muenst, Soysal, Tzankov, & Hoeller, 2015). Excessive phosphorylation and inactivation of TTP is thought to contribute to tumorigenesis (Coelho et al., 2017; Suswam et al., 2013; Tran et al., 2016).

TTP is efficiently dephosphorylated by PP2A (Mahtani et al., 2001; Sun et al., 2007). The dynamic equilibrium between MK2-mediated phosphorylation (inactivation) and PP2A-mediated dephosphorylation (activation) of TTP allows precise regulation of rates of degradation of pro-inflammatory mRNAs via the MAPK p38 - MK2 pathway (Clark & Dean, 2016; Gaestel, 2013; Kratochvill et al., 2011; O'Neil et al., 2018; Sedlyarov et al., 2016; Smallie et al., 2015; Tiedje et al., 2016). Activation of TTP may help to explain observations that PP2A inhibitors increased expression of pro-inflammatory genes by stabilizing mRNA rather than (or as well as) activating transcription (Cornell et al., 2009; Mahboubi, Young, & Ferreri, 1997; Sun et al., 2007; Sun et al., 2017; Sung, Walters, & Fu, 1992; Yoza, Wells, & McCall, 1998). Increased PP2A activity in ageing B cells was implicated in enhanced TTP-mediated degradation of *Tcf3* mRNA, which encodes an important regulator of B cell function (Frasca et al., 2007; Frasca et al., 2010; Frasca, Landin, Riley, & Blomberg, 2008). Miscontrol of TTP function may therefore contribute to age-related deficits of B cell function.

The expression of pro-inflammatory mediators in airway epithelial cells was decreased by both FTY720 and its non-phosphorylatable analogue AAL(s), in a TTP-dependent manner (Rahman et al., 2015; Rahman et al., 2016). We therefore tested the hypothesis that PADs exert anti-inflammatory effects via the activation of TTP (Ross et al., 2017). The apolipoprotein E-derived peptide COG1410 decreased the expression of TNF and CXCL2 in wild type macrophages, but had no significant effect on the weak expression of these inflammatory mediators in *Zfp36aa/aa* macrophages, in which TTP is already constitutively dephosphorylated and active. In parallel, COG1410 decreased the expression of wild type TTP protein but did not influence the expression of the non-phosphorylatable mutant TTP protein. COG1410-mediated suppression of TNF expression was dependent on an intact TTP binding site in the *Tnf* 3’ UTR (Ross et al., 2017). All of these observations suggest that COG1410 exerts anti-inflammatory effects *in vitro* by promoting the dephosphorylation and activation of TTP. Both COG1410 and AAL(s) exerted therapeutic effects in an experimental model of rheumatoid arthritis (Ross et al., 2017).

These findings constitute proof of principle that PADs may exert anti-inflammatory effects at least in part via the activation of TTP. However, we still know relatively little about the dephosphorylation side of the TTP equilibrium. *In vitro* dephosphorylation of TTP was performed using a commercial preparation that consists mainly of catalytic and scaffolding subunits (Mahtani et al., 2001; Sun et al., 2007). The involvement of B subunits in the targeting of TTP for dephosphorylation is so far unexplored. It is not known where in the cell the process takes place. It is also unclear whether TTP dephosphorylation is constitutive or regulated, for example via changes in expression of the relevant B subunit(s) or their assembly into holoenzymes.

## 5 Neuro-inflammation and neuro-degeneration

### 5.1 Introduction

Neuro-inflammation is characterized by functional compromise of the blood–brain barrier (BBB), infiltration of the brain by leukocytes that are otherwise normally excluded, and aberrant activation of glial (that is, non-neuronal) cells (Laurent, Buee, & Blum, 2018; Skaper, Facci, Zusso, & Giusti, 2018; Stephenson, Nutma, van der Valk, & Amor, 2018). Glial cells in the brain include oligodendrocytes, whose principal function is to generate and maintain the insulating myelin sheaths of neurons; astrocytes, which biochemically and structurally support neuronal and BBB function; and microglia, resident myeloid cells which serve homeostatic functions in the central nervous system (CNS), participate in synaptic remodelling and mediate inflammatory responses to injury or infection. Neuro-inflammation is very often accompanied by neuro-degeneration, causing various degrees of motor, sensory, cognitive and affective symptoms. Neuro-inflammation may be either cause or consequence of neuro-degeneration, and the causal relationships between these two intimately connected, mutually reinforcing processes can be difficult to establish. Here we will focus on two very distinct neuro-inflammatory and neuro-degenerative conditions in which PP2A has gained attention for quite different reasons.

### 5.2 Alzheimer’s disease

The principal cause of dementia is the progressive neuro-degenerative condition known as Alzheimer’s disease (AD). A defining histological feature of AD is the presence of extracellular plaques composed of insoluble aggregates of amyloid β peptides, and intracellular neurofibrillary tangles (NFT) composed of aggregates of tau protein. Tau is a microtubule-associated protein possessing three to four C terminal tubulin-binding motifs (according to which splice isoform of the primary transcript is expressed). It regulates synaptic plasticity of neurons by controlling microtubule dynamics, and the formation and stability of neurotransmitter-activated signaling complexes at the cell surface. According to the tau hypothesis, the formation of NFTs is the key pathogenic process driving neuronal dysfunction and death, not only in AD but also in other neurodegenerative conditions collectively known as tauopathies (Iqbal et al., 2010; Sabbagh & Dickey, 2016; Sontag & Sontag, 2014; Voronkov, Braithwaite, & Stock, 2011; Ward, Himmelstein, Lancia, & Binder, 2012). In this scenario, amyloid plaque formation in AD is secondary to NFT-mediated neurodegeneration, although it may also exacerbate pathology by causing neurodegeneration in its own right, promoting the activation of resident glial cells and the recruitment of inflammatory monocytes from the circulation. Interactions of tau with tubulin are regulated by phosphorylation of sites that flank the tubulin-binding domain. Several kinases have been implicated in tau phosphorylation; notably glycogen synthase kinase 3β, cyclin-dependent kinase 5 and extracellular signal-regulated kinase 2. Hyper-phosphorylation leads to impairment of the normal physiological functions of tau, its oligomerization, and ultimately the formation of NFTs. One possible approach to treatment of AD is to inhibit kinases responsible for tau hyperphosphorylation. At the time of writing, clinical trials of a glycogen synthase kinase 3β inhibitor are ongoing (del Ser et al., 2013; Lovestone et al., 2015; Medina, Garrido, & Wandosell, 2011).

Approximately 70% of tau-dephosphorylating activity in neurons can be attributed to PP2A holoenzyme that contains the specificity subunit PPP2R2A (Liu, Grundke-Iqbal, Iqbal, & Gong, 2005; Martin et al., 2013; Sontag et al., 1999). The structure of this holoenzyme complex has been solved, providing important insights into the mechanism of targeting tau for dephosphorylation (Xu, Chen, Zhang, Jeffrey, & Shi, 2008). As previously remarked, the incorporation of members of the PPP2R2 family into the PP2A holoenzyme is dependent on carboxy terminal methylation of the catalytic subunit PPP2CA, and sensitive to perturbations that inhibit this post-translational modification. Tau hyper-phosphorylation and some AD-like features can be caused or exacerbated by experimental manipulation of PP2A activity, for example using PP2A-inhibiting chemicals (Gong, Wang, Iqbal, & Grundke-Iqbal, 2003; Kamat, Rai, & Nath, 2013), over-expression of DNA tumor virus T antigens (Sontag, Nunbhakdi-Craig, Lee, Bloom, & Mumby, 1996) or PP2A inhibitory proteins (Wang, Blanchard, Tung, Grundke-Iqbal, & Iqbal, 2015), introduction of a dominant negative catalytic subunit (Deters, Ittner, & Gotz, 2009) or inhibition of PP2CA methylation (Sontag, Nunbhakdi-Craig, & Sontag, 2013). Conversely, PP2A activity is reportedly reduced by about 50% in AD-affected CNS tissue. As reviewed in detail elsewhere (Sontag & Sontag, 2014; Voronkov et al., 2011), several different mechanisms of PP2A dysfunction have been described in AD. These include: decreased expression of the PPP2R2A subunit; increased expression or altered subcellular localization of endogenous PP2A inhibitor proteins; decreased expression, reduced Leu309 methylation. Increased Tyr307 phosphorylation of the catalytic subunit of PP2A has also been described, although as noted above these observations need to be interpreted with caution.

Evidence of PP2A dysfunction has prompted researchers to investigate therapeutic responses to PADs in experimental models of AD. Cognitive impairment driven by amyloid β aggregates was reduced following treatment of animals with the novel PP2A-activating compound EHT (Asam et al., 2017; Basurto-Islas et al., 2014) or FTY720 (Asle-Rousta, Kolahdooz, Dargahi, Ahmadiani, & Nasoohi, 2014; Asle-Rousta, Kolahdooz, Oryan, Ahmadiani, & Dargahi, 2013; Hemmati et al., 2013). Sodium selenate, which increases PP2A activity, reduced cognitive impairment in a PP2A-dependent manner in AD models based on dysregulated tau phosphorylation (Corcoran et al., 2010; van Eersel et al., 2010). A copper ionophore, Cu^II^(gtsm), conferred functional protection in both amyloid β- and tau-mediated models of AD (Crouch et al., 2009; McKenzie-Nickson et al., 2018). This compound increased the expression of the scaffolding subunit PPP2R1A in mouse brain, but increased PP2A activity remains to be demonstrated. Mechanistic studies of these compounds have focused on neurotoxicity. For example, FTY720 antagonised amyloid β-induced neuronal death (Aytan et al., 2016; Doi et al., 2013; Hemmati et al., 2013; Joshi et al., 2017; Ruiz et al., 2014) in a manner that depended on sphingosine kinase activity, and was opposed by S1P receptor antagonists (Asle-Rousta et al., 2014; Joshi et al., 2017). These findings suggest that phosphorylated FTY720 may exert beneficial effects on neurons via agonistic rather than antagonistic action at S1PR1. In the context of AD, there remains a gap in knowledge about effects of FTY720 and other putative PADs on glial cells.

### 5.3 Multiple sclerosis and other neuro-inflammatory disorders

MS is a chronic immune-mediated inflammatory disease in which auto-reactive lymphocytes participate in destruction of the insulating myelin sheaths of neurons, causing neurological dysfunction, pain and physical disability (Brinkmann et al., 2010; Chaudhry et al., 2017; Mehling et al., 2011). FTY720 reduced neuro-inflammation and neurological deficits in experimental autoimmune encephalopathy (EAE), a widely used animal model of MS (Brinkmann et al., 2002; Kataoka et al., 2005). Following successful clinical trials, it was licensed for use in the relapsing remitting form of MS. Its annual sales now exceed two billion dollars, and it is under consideration as a possible treatment for other CNS pathologies such as cerebral ischemia, traumatic CNS injury, epilepsy and Parkinson’s disease (Huwiler & Zangemeister-Wittke, 2018). According to the orthodox narrative, FTY720 exerts anti-inflammatory effects by blocking lymphocyte egress from lymph nodes, causing peripheral lymphopenia and interrupting traffic to the CNS of auto-reactive T cells (Brunkhorst, Vutukuri, & Pfeilschifter, 2014; Chun & Hartung, 2010; Cohen & Chun, 2011; Mehling et al., 2011). However, in MS, other neuroinflammatory pathologies and their experimental models, there is often a poor correlation between lymphopenia and the beneficial effects of FTY720 and related compounds, suggesting that additional mechanism of action are likely to be important (Choi et al., 2011; Foster et al., 2007; Healy, Michell-Robinson, & Antel, 2015; La Mantia et al., 2016; Rothhammer et al., 2017; Serdar et al., 2016). In fact, beneficial effect of FTY720 have been demonstrated in a variety of CNS resident cells, including astrocytes, microglia, oligodendrocytes and vascular endothelial cells (Groves, Kihara, & Chun, 2013; Miron, Schubart, & Antel, 2008). It remains a subject of lively debate, whether these are mediated by agonistic or antagonistic effects of phosphorylated FTY720 on S1P receptors. The possibility that activation of PP2A contributes to therapeutic effects of FTY720 is not often considered.

In a variant EAE model, astrocyte-specific deletion of the gene encoding S1PR1 both reduced the severity of disease and ablated therapeutic responses to FTY720 (Choi et al., 2011). These observations suggest that S1P mediates pathogenic activation of astrocytes via S1PR1, and that FTY720 exerts protective effects via blockade of the S1P-S1PR1 axis. However, other researchers implicated S1PR3 as a mediator of pro-inflammatory responses of astrocytes to S1P. This receptor signals via G_α12/13_ to activate the small GTPase RhoA and the transcription factor NF-κB (Dusaban et al., 2013; Dusaban, Chun, Rosen, Purcell, & Brown, 2017), a mechanism essentially similar to that previously described in vascular endothelial cells (Fernandez-Pisonero et al., 2012; Keul et al., 2011; Sanchez et al., 2007; Skoura et al., 2011). Activation of NF-κB is essential for pro-inflammatory gene expression by astrocytes (Brambilla et al., 2005; van Loo et al., 2006). Global effects of FTY720 on the astrocyte transcriptome were consistent with inhibition of NF-κB activation (Rothhammer et al., 2017), and indeed FTY720 impaired the activation of NF-κB in astrocytes following stimulation with S1P or LPS (Colombo et al., 2014; Rothhammer et al., 2017). But here the links between FTY720 and astrocyte inflammatory gene expression seem to break down. S1PR3 is not thought to be a target of functional antagonism by FTY720 (Brinkmann et al., 2002; Graler & Goetzl, 2004); and S1PR1, which signals primarily via G_i_, does not have a well-established mechanism of coupling to NF-κB (Siehler & Manning, 2002). Therefore it is unclear whether inhibition of NF-κB is the main mechanism by which FTY720 decreases expression of inflammatory mediators in astrocytes, and if it is, how this inhibition is achieved.

In MS and other neuro-inflammatory pathologies microglia, the resident myeloid cells of the CNS, adopt a pro-inflammatory activation state that is sometimes referred to as “M1” (Baufeld, O'Loughlin, Calcagno, Madore, & Butovsky, 2018; Mammana et al., 2018; Orihuela, McPherson, & Harry, 2016; Skaper et al., 2018; Xu, He, & Bai, 2016). This is accompanied by a metabolic shift in favour of aerobic glycolysis, which can be detected by positron emission tomography on the basis of increased uptake of a labelled glucose analogue. FTY720 decreases CNS glycolytic activity in MS and its experimental models, suggesting a direct anti-inflammatory effect on microglia (Airas et al., 2015; Anthony, Sibson, Losey, Meier, & Leppert, 2014; Sucksdorff et al., 2017). FTY720 inhibited the expression of the pro-inflammatory cytokines TNF and IL-1β in activated primary microglia, microglial cell lines and microglia-containing organotypic cultures (Das et al., 2017; Jackson, Giovannoni, & Baker, 2011; Noda, Takeuchi, Mizuno, & Suzumura, 2013; Qin et al., 2017). At the transcriptome level, FTY720 significantly reduced the expression of several hundred genes in LPS-activated primary microglia (Das et al., 2017). FTY720-induced changes of gene expression were consistent with skewing from pro-inflammatory, “M1” to pro-reparative “M2” microglia (Qin, Fan, et al., 2017), which may contribute to neuro-protection (Giunti, Parodi, Cordano, Uccelli, & Kerlero de Rosbo, 2014; Kanazawa, Ninomiya, Hatakeyama, Takahashi, & Shimohata, 2017). There is some evidence that S1P-S1PR1 signaling promotes pro-inflammatory gene expression in microglia (Gaire et al., 2018), and that FTY720 exerts anti-inflammatory effects by disrupting this axis (Noda et al., 2013). However, the mechanism of anti-inflammatory action of FTY720 in microglia has not been characterized in detail, and possible involvement of PP2A has not been investigated.

Another myeloid cell type, the dendritic cell (DC), is critically involved in the dysregulated immune responses that underlie MS (Luessi, Zipp, & Witsch, 2016; Mishra & Yong, 2016). Several groups have demonstrated inhibitory effects of FTY720 on DC traffic, which could contribute to beneficial effects of the drug (Gollmann et al., 2008; Han et al., 2015; Idzko et al., 2006; Lan et al., 2008; Maeda et al., 2007; Reines et al., 2009). In addition, FTY720 impairs the function of DCs, reducing their capacity to secrete pro-inflammatory cytokines and stimulate T cell proliferation (Durafourt et al., 2011; Han et al., 2015; Heng et al., 2010; Idzko et al., 2006; Luessi et al., 2015; Muller et al., 2005; Ruger et al., 2014; Thomas et al., 2017; Zeng et al., 2012). In many of these studies FTY720 reduced the expression of IL-12 by activated DCs, which is consistent with impairment of Th1 responses. However, FTY720 was also shown to impair DC-mediated Th2 responses in an experimental model of asthma (Idzko et al., 2006). Intriguingly, FTY720 was superior to phospho-FTY720 as an inhibitor of DC-mediated cytokine expression (Thomas et al., 2017) or T cell activation (Ruger et al., 2014), an observation that is difficult to reconcile with an S1PR-mediated mechanism. PP2A was tentatively identified as a mediator of the effect of FTY720 on DCs (Ruger et al., 2014), but no detailed mechanism has yet been described.

Several groups have shown that FTY720 exerts protective effects and slows functional decline in animal models of Parkinson’s disease (PD) (Motyl, Przykaza, Boguszewski, Kosson, & Strosznajder, 2018; Ren et al., 2017; Zhao et al., 2017). One study implicated agonistic effects of FTY720 on S1PR1 as a therapeutic mechanism (P. Zhao et al., 2017). However, impairment of PP2A function has been noted in PD and its experimental models (Braithwaite, Voronkov, Stock, & Mouradian, 2012; Hua et al., 2015; Park et al., 2016; Taymans & Baekelandt, 2014; Wu et al., 2012). One group has explored therapeutic effects of FTY720 analogues that activate PP2A but cannot be phosphorylated and do not cause lymphopenia (Segura-Ulate, Belcher, Vidal-Martinez, Vargas-Medrano, & Perez, 2017; Vargas-Medrano et al., 2014; Vidal-Martinez et al., 2016). Other investigators demonstrated protective effects of the PP2A-activating drug EHT in two different models of PD (Lee et al., 2011; Lee, Jeong, et al., 2013).

FTY720 reduced lesion size, microglial activation and neurological deficits in experimental models of stroke or intracerebral hemorrhage (Brait, Tarrason, Gavalda, Godessart, & Planas, 2016; Czech et al., 2009; Hasegawa, Suzuki, Sozen, Rolland, & Zhang, 2010; Kraft et al., 2013; Nazari, Keshavarz, Rafati, Namavar, & Haghani, 2016; Rolland 2nd et al., 2011; Rolland et al., 2013; Wei et al., 2011). These preclinical findings prompted a small clinical trial, which reported promising therapeutic effects of FTY720 in stroke (Fu et al., 2014), and are being followed up in a larger trial (Fu et al., 2014). The beneficial effects of FTY720 in stroke and experimental models of brain ischemia were linked to prevention of BBB dysfunction (Fu, Zhang, et al., 2014; Kraft et al., 2013; Rolland et al., 2013; Wei et al., 2011). However, the role of S1PR1 in ischemic brain injury remains controversial, with reports of protective effects of both S1PR1-selective agonists and antagonists (Brait et al., 2016; Gaire et al., 2018; Hasegawa, Uekawa, Kawano, Suzuki, & Kim-Mitsuyama, 2017; Sun et al., 2016). To some extent these discrepancies may be explained by differences between the experimental models used, or mixed agonist/antagonist properties of the compounds used to manipulate S1PR1 signaling. The possible involvement of PP2A in therapeutic effects of FTY720 has not been explored in these contexts. However, protective effects of PP2A-activating peptides have been demonstrated in experimental models of ischemic or physical brain injury (Cao et al., 2016; Hoane, Kaufman, Vitek, & McKenna, 2009; Kaufman et al., 2010; Laskowitz et al., 2012; Pang et al., 2017; Qin et al., 2017; Tukhovskaya, Yukin, Khokhlova, Murashev, & Vitek, 2009; Wu et al., 2016). In some of these studies, protection of neurological function was accompanied by prevention of BBB dysfunction (Cao et al., 2016; Pang et al., 2017; Qin, You, et al., 2017), suggesting at least some degree of overlap between therapeutic effects of FTY720 and PADs.

## 6 Remaining questions

In only a little more than a decade, PP2A has emerged as an exciting therapeutic target in several types of cancer. This approach is being actively explored by so many research groups in academia and the pharmaceutical industry, the first clinical trials may not be far off. Dysregulation of tau phosphorylation in Alzheimer’s disease and other CNS pathologies has placed PP2A in the therapeutic spotlight for slightly longer. There remain significant obstacles to the translation of basic research discoveries in this area, as discussed eloquently elsewhere (Sontag & Sontag, 2014; Taleski & Sontag, 2018). Many of the same concerns arise when PP2A is considered as a possible therapeutic target in inflammatory diseases. This is a comparatively new concept, and several important questions remain to be answered.

### 6.1 Is PP2A activity impaired in inflammatory diseases? And does it matter?

Reactive oxygen species (ROS) are often abundant at sites of inflammation (McGarry, Biniecka, Veale, & Fearon, 2018). Although some observations are contradictory, there is general consensus that PP2A activity is impaired by ROS and other radicals, for example via oxidation of critical thiol groups in the reactive site (Elgenaidi & Spiers, 2019; Jin Jung et al., 2013; Raman & Pervaiz, 2019; Tan, Shavlakadze, Grounds, & Arthur, 2015). Prolonged exposure of mice to cigarette smoke increased the expression of the PP2A inhibitor CIP2A, impairing PP2A function and increasing the expression of metallo-proteinases (Nath et al., 2018). Increased expression of CIP2A may contribute to sustained inflammation and tissue damage in chronic obstructive pulmonary disease (Nath et al., 2018). Elevated CIP2A expression has also been reported in rheumatoid arthritis, where it is thought to contribute to the abnormal resistance of synovial fibroblasts to apoptosis (Lee et al., 2012; Lee, Jeong, et al., 2013). As remarked above, pro-inflammatory or immune-stimulatory agonists may also influence the expression of B subunits of PP2A (Breuer et al., 2014), suggesting a potential mechanism for fine-tuning of PP2A activity in the context of inflammatory or immune responses.

Overall, remarkably little is known about the regulation or dysregulation of PP2A in inflammatory disease. The knowledge gap needs to be filled, but it is not necessarily fatal to the concept of therapeutic targeting of PP2A. A few proof-of-principle experiments have already shown that PP2A-activating compounds exert therapeutic effects on experimental inflammatory pathologies (Collison et al., 2013; McHugh et al., 2016; Nair et al., 2017; Ross et al., 2017). Similar effects might be seen in human inflammatory disease, provided that PP2A is not absent or quantitatively and irreversibly inactivated (both of which seem unlikely). The expression of PP2A inhibitory proteins at sites of inflammation is one important issue that requires further attention, since many PADs function by interrupting interactions between these proteins and PP2A itself.

### 6.2 Are all PADs likely to be similar in their effects?

Several PADs specifically target the inhibitor protein SET; some directly target the scaffolding subunit PPP2R1A to cause allosteric activation of the holoenzyme complex; others promote methylation of the catalytic subunit PPP2CA; and direct catalytic inhibitors of the PP2A inhibitor PME-1 are also under development (Bachovchin et al., 2010). As discussed above (3.2), differences in mechanism of action of these compounds are likely to result in different cellular responses to PADs, although to our knowledge this has not yet been demonstrated. Molecules described as PADs also belong to completely different chemical classes, so that their off-target profiles are very likely to differ. Again, this aspect has not yet been systematically studied.

### 6.3 Can any therapeutic effects of FTY720 be ascribed to PP2A activation?

FTY720 has a strange double existence in biomedical research. In the field of oncology, FTY720 is well established as a PP2A-activating compound, and it is the foundation of efforts to therapeutically target PP2A. In the fields of inflammation, neuro-inflammation and neuro-degeneration, FTY720 is widely discussed as a therapeutic tool. Although it is increasingly difficult to explain the beneficial effects of FTY720 solely in terms of impaired leukocyte traffic, the possible importance of PP2A activation is very often overlooked. There are adequate experimental tools to resolve this conundrum. Non-canonical effects of FTY720 should be independent of sphingosine kinase activity, and retained by analogues that cannot be phosphorylated. In this context it is worth noting that AAL(s) failed to reduce symptom severity in EAE (Brinkmann et al., 2002), an important finding that argues against a major role of PP2A as a mediator of protective effects of FTY720 in this experimental model. It would be valuable to confirm this finding, and to extend it by testing effects in EAE of PADs that are unrelated to FTY720. In principle, selective agonists and antagonists of S1P receptors should also be useful tools with which to rule in or out the involvement of S1P-mediated inside out signaling. However, mixed agonistic-antagonistic effects of such compounds may lead to increased confusion rather than clarity.

### 6.4 Are PADs likely to be well tolerated?

The influence of PP2A on cell biology is both broad and deep, giving rise to very strong concerns about the desirability of indiscriminate PP2A activation. This is particularly true of chronic but not directly life-threatening inflammatory conditions, in which patients may be exposed to treatments for decades. Most of the pharmacological development of PADs has taken place in the cancer domain, where the balance of benefit against risk is different. Several PADs have demonstrated *in vitro* cytotoxic effects on transformed but not on normal cells, and some have demonstrated favorable safety profiles *in vivo*. But much of the toxicology work has been done in relatively short exposure settings, and most of it in rodent models. There remains a great deal of work to show whether long term treatment with PADs will be safe in humans.

It may be possible to derive some safety information from drugs that have already been used extensively in man. The most obvious example is FTY720, the side effects of which are well described and broadly manageable (Jeffery, Rammohan, Hawker, & Fox, 2016; Ward, Jones, & Goldman, 2014). The methylxanthine drug theophylline and the β adrenergic agonists salmeterol and formoterol have been widely used in the treatment of asthma and chronic obstructive pulmonary disease, and have also been described as activators of PP2A (Hatchwell et al., 2014; Kobayashi et al., 2012; Patel et al., 2016; Pullar, Chen, & Isseroff, 2003; Rahman et al., 2016; Sokulsky et al., 2016). On this basis it could be argued that PADs are likely to be tolerated.

### 6.5 Is precise targeting preferable to indiscriminate targeting of PP2A?

This is closely connected to some of the preceding questions, and equally difficult to answer at present. If long-term, global stimulation of PP2A activity incurs significant health risks, then it may ultimately be possible to mitigate these risks by targeting specific holoenzyme complexes, using compounds that interact with the holoenzyme in a manner dependent on its B subunit. Again, we emphasise the unhelpfulness of discussing PP2A as if it is a single entity. Instead we need to understand the expression, substrate specificity and cellular functions of specific PP2A holoenzyme complexes (effectively meaning the expression, specificity and function of B subunits). In this respect, there is so far relatively little information in the context of inflammation, and it remains difficult to measure or manipulate the activity of PP2A in a precise way.

## 7 Conclusion

There is considerable evidence that PP2A plays an important role in constraining inflammation, and a strong suggestion that some licensed drugs may exert therapeutic effects at least in part via PP2A activation. However, the biology of PP2A is so complex that it may never be possible to fully predict harmful and beneficial effects of PADs. Clinical trials of these compounds are likely to take place first in the cancer field, where the evidence base is strongest and the balance of risk to benefit most favourable to experimental medicine. If and when such trials do take place, it would make good sense to monitor potential anti-inflammatory and immune-modulatory effects. And if the safety profiles of PADs are found to be acceptable, there is an argument for rapidly undertaking clinical trials in the context of inflammatory disease.

## Conflict of interest statement

ARC asserts no conflict of interest. Mount Sinai School of Medicine has filed patents on PP2A-activating drugs, including DBK-1154, on behalf of MO.

## Acknowledgements

ARC’s research is supported by Project Grant G0800207 from the Medical Research Council UK, Programme Grant 21802 from Versus Arthritis, and the Rheumatoid Arthritis Pathogenesis Centre of Excellence (RACE) award from Versus Arthritis.
